# Impacts of corn intercropping with soybean, peanut and millet through different planting patterns on population dynamics and community diversity of insects under fertilizer reduction

**DOI:** 10.3389/fpls.2022.936039

**Published:** 2022-10-18

**Authors:** Likun Li, Ruichuan Duan, Runzhao Li, Yan Zou, Jiawen Liu, Fajun Chen, Guangnan Xing

**Affiliations:** ^1^Department of Entomology, College of Plant Protection, Nanjing Agricultural University, Nanjing, Jiangsu, China; ^2^Soybean Research Institute & MARA National Center for Soybean Improvement & MARA Key Laboratory of Biology and Genetic Improvement of Soybean & National Key Laboratory for Crop Genetics and Germplasm Enhancement & Jiangsu Collaborative Innovation Center for Modern Crop Production, Nanjing Agricultural University, Nanjing, China

**Keywords:** corn, intercropping type, planting pattern, fertilizer reduction, population dynamics, community diversity, insect pest management

## Abstract

Corn is one of the key grain crops in China and the excessive use of chemical fertilizers and pesticides seriously damages the ecological environment in fields. To explore a more scientific and reasonable way to plant corn and simultaneously reduce the overuse of chemical fertilizers and pesticides, the impact of corn intercropping with soybean, peanut, and millet, respectively, through five planting patterns, including three intercropping patterns (2 corn rows to 2, 3 and 4 rows of soybean/peanut or 2, 4 and 6 millet rows, respectively) and two monoculture patterns of corn and soybean, peanut or millet under normal (600 kg/ha) and reduced (375 kg/ha) levels of NPK (N:P_2_O_5_:K_2_O = 15:15:15) fertilization on the population abundance and community diversity of insects, leaf nutrients, and induced plant hormones jasmonic acid (JA) and salicylic acid (SA) was studied in 2018 and 2019. The results showed that the insect community indexes of the species number (*S*), the diversity index (*H*), and the uniformity index (*E*) generally increased under intercropping and were significantly higher than those under corn monoculture. The prevalence of Asian corn borer (*Ostrinia furnacalis*) on the intercropping corn plants decreased by based on the average of seven surveys per year for each treatment 2.9 to 17 heads per 30 plants compared with that on the monoculture corn plants. The number of natural enemy insect species on corn plants under intercropping was significantly higher than that under corn monoculture. That is, intercropping may decrease the population of Asian corn borers by increasing *S*, *H*, *E*, and natural enemy insect species (NEI). Moreover, intercropping type and fertilizer level significantly affected corn leaf nutrient contents. Compared with the normal fertilizer level, fertilizer reduction significantly reduced the foliar contents of amino acids, soluble protein, and soluble sugar in corn plants. In addition, corn–soybean and corn–peanut intercropping significantly increased the three nutrient contents in corn leaves compared with corn monoculture. In terms of corn nutrients, intercropping could compensate for the effects of fertilizer reduction. The foliar content of JA in corn–soybean intercropping was significantly higher than in corn monoculture. Under corn–soybean and corn–peanut intercropping, SA was significantly lower than under corn monoculture. Overall, intercropping, not fertilizer reduction, can significantly increase insect community diversity while reducing the population abundances of the key insect pest species on corn plants. Intercropping reduced the SA content, increased amino acids and thus reduced the susceptibility of corn to the pest insects.

## Introduction

Intercropping refers to two or more crops that are closely mixed in planting in the same farmland system during the whole or part of a growing season (Vandermeer, 1989; Willey, 1990). It has a history of thousands of years in China (Lichtfouse, 2009). As the most representative planting system, intercropping has been widely used in the agro-ecosystem because it can enhance the complementarity and utilization of nutrients and environmental resources (Wang et al., 2007; Bargaz et al., 2017; Duchene et al., 2017). By exploiting complementarities among intercrops to improve resource use efficiency and crop yields, it is possible to reduce pressure on land and water sources (Singh et al., 2019; Chen et al., 2019; Du et al., 2019). Therefore, intercropping is called the “New Green Revolution” of sustainable agricultural intensification (Martin-Guay et al., 2018). In addition, intercropping can not only increase crop yield but also improve soil fertility (Gong et al., 2019), which is beneficial to the continuous increase of farmland yield (Lithourgidis et al., 2011; Yu et al., 2019). Compared with monoculture, intercropping can also reduce the occurrences of diseases and insect pests (Horwith, 1985; Vandermeer, 1989; Zhu et al., 2000; Machado, 2009; Lithourgidis et al., 2011; Boudreau, 2013; Zhang et al., 2019). It was found that corn–peanut intercropping increased the population abundance of ladybugs and reduced the population density of peanut aphids (Ju et al., 2019). It has also been found that the prevalence of tortoise ladybugs increased, and the population abundance of cotton aphids, *Aphis gossypii,* decreased under corn–cotton intercropping (Ouyang et al., 2020). The mechanism of pest reduction in cereal-legume intercropping can be summarized into three points: (1) intercropping can reduce the chances of pests finding suitable hosts because chemical and visual cues and stimuli reduce (Songa et al., 2007); (2) intercropping can decrease the likelihood that larvae will migrate to a suitable host, as larvae can also be transferred from one plant to another (Wale et al., 2007); and (3) intercropping can attract the natural enemies of insect pests, thus reducing the invasion of cereal crops (Songa et al., 2007).

Nitrogen-fixing crops (NFCs) such as soybeans and peanuts are often used as intercrops in intercropping patterns. The NFCs can fix N from the atmosphere and improve soil fertility, provide nutrients for plant growth, and change the level of other nutrients in the soil (Hui et al., 1999, 2003). The contents of N, C, P, and organic matter in soil were significantly increased by Acacia (Lorenzo et al., 2010). *Robinia pseudoacacia* was able to fix N, and the N content in the soil increased to 1.3–3.2 times (Rice et al., 2004). The accumulation of nutrients from NFCs described above or from fertilizers can affect insect occurrence in the field. The increase in N-fertilizer usage could inhibit the occurrence of potato pests to some extent (Fragoyiannis et al., 2001). The rise in N-fertilizer usage also increased the tightness of corn husks, thus reducing the infection of corn earworms (Klostermeyer, 1950). In addition, with the increase in N content in plant tissues, the population densities of sucking insect pests increased while the number of chewing insect pests decreased (Pimentel and Warneke, 1989). Higher N-fertilizer levels could promote the development and fertility of cotton aphids (Awmack and Leather, 2001; Cisneros and Godfrey, 2001). Based on previous studies, it can be found that fertilizer level indirectly affects insect population occurrence mainly through affecting crop phenotype or nutrient content. Different species of insects have different responses to fertilizer levels. These are either beneficial or harmful.

Moreover, literature has shown that fertilization can enhance the resistance of plants against herbivorous insects (Smith, 1978; Rathjens and Funk, 1986; Neely et al., 1987; Caldwell and Funk, 1999). From this research, it is known that nutrients can influence the defense mechanisms of plants. For the plant defense mechanisms, JA (Odonnell et al., 1996; Farmer et al., 2003) and SA (Glazebrook, 2005) play essential roles in signal transduction. Plant nutrient content has a particular influence on the insect resistance of plants, and the total N is considered the most critical nutrient (Mattson, 1980; Scriber, 1984; Slansky and Rodriguez, 1987).

In the present study, the impact of corn intercropping with NFC crops (including soybean and peanut) and a non-NFC crop (i.e., millet), respectively, through five planting patterns, including three intercropping patterns and two monoculture patterns of corn and soybean, peanut or millet under normal and reduced levels of NPK fertilizer, on the population dynamics of key herbivorous insects and community diversity of insects was researched. Leaf nutrients and induced defense were bioassayed to explore the plant nutrients, defense, and the resistance mechanism of corn crops against insect pests in the intercropping agroecosystem under fertilizer reduction.

## Materials and methods

### Experimental location

The experiments were conducted in the Nanxin Village, Jiyang district, Jinan city of Shandong Province of China (36°58’N, 117°13′E). The local climate conditions during the test period are shown in Table 1. The test period was from June to October each year.

**Table 1:** Meteorological data of Jiyang district, Jinan city of Shandong Province of China in 2018 and 2019.

Year	Meteorological factor	January	February	March	April	May	June	July	August	September	October	November	December
2018	Average temperature (°C)	−2.5	0.3	9.7	16.0	21.1	26.5	28.6	27.2	20.8	13.2	7.3	−0.9
	Precipitation (mm)	2.7	0.1	19.9	72.5	104.6	201.2	97.2	246.7	24.5	50.9	5.5	3
	Relative humidity (%)	55	45	56	57	67	61	80	84	76	69	75	58
2019	Average temperature (°C)	−1.4	0.8	10.0	13.8	21.9	27.3	28.0	25.2	21.5	14.2	7.7	0.7
	Precipitation (mm)	0	4	2.1	32.2	6.4	31.7	66.1	196.5	15.3	17.6	21.5	9.4
	Relative humidity (%)	54	62	47	61	49	53	69	78	75	72	69	72

Data from Jiyang Statistical Yearbook for 2018 and 2019 (Jiyang Investigation Team From the National Bureau of Statistics, 2018, 2019).

### Crops cultivars

In the present experiment, four different grain crops were used, including corn, millet, soybean, and peanut. The four grain crops selected for the present study are frequently used for intercropping in Northern China. Among them, soybean (*cv.* Xindou 1; provided by Jinan Zhaohui Seed Industry Co., Ltd. from Jinan City of Shandong Province of China) and peanut (*cv.* Huayu 22; provided by the Shandong Peanut Research Institute at Qingdao City of Shandong Province of China) are nitrogen-fixing crops (NFCs). Corn (*cv.* Liangyu 99; provided by Dandong Denghai Liangyu Seed Industry Co., Ltd. from Dandong City, Liaoning Province of China) and millet (*cv.* Lazy Valley No. 3; provided by the Lutong Seed Industry Co., Ltd. from Yongnian County, Hebei Province, China) are non-NFC crops (Li et al., 2022).

### Field experiment setup

In the present study, there were three intercropping types (i.e., corn–soybean/peanut/millet intercropping), five planting patterns (including three planting patterns of 2 corn rows to 2, 3, and 4 rows of soybean/peanut, respectively, and to 2, 4, and 6 rows of millet, respectively, and two monoculture patterns of corn and soybean/peanut/millet), and two fertilizer levels (i.e., normal (600 kg/ha) and reduced (375 kg/ha) levels of NPK (N: P2O5: K2O = 15: 15: 15; fertilizer), and a completely random design with three repetitions. The row and hill spacing of corn, soybean, and peanut were 0.8 m and 0.2 m, respectively. However, the spacing of millet was 0.2 and 0.05 m respectively, and the spacing of two millet rows to two neighboring millet rows was 0.8 m. The spacing of the corn row to the row of soybean, peanut, and millet was 0.8 m. The planting density of corn, soybean, peanut, and millet was approximately 6.25, 12.5, 12.5, and 40 plants/m^2^, respectively. Corn and millet had one plant in each hill, peanut and soybean had two plants in each hill. The length and width of each plot were 28.8 and 15.5 m, respectively, and 1.0 m spacing with the neighbor plots (Li et al., 2022). The arrangement figure of each treatment and the effects of these factors on corn biomass and yield have been published (Li et al., 2022). Four crops were sown on June 16 of 2018 and 2019, respectively. The planting cultivation adopted local field management measures, including one-time irrigation and one-time spraying of herbicides before planting, and no insecticides were sprayed during the whole growing seasons of 2018 and 2019 (Li et al., 2022). All crops were harvested in October each year.

### Leaves sampling and foliar chemicals bioassay

At the heading stage, ten corn plants were randomly selected in each plot and the first leaf on top of each plant was cut and immediately frozen in liquid nitrogen with three replications and then taken to the laboratory to extract and quantify the content of plant hormones JA and SA in the leaves as per the test method of Wu et al. (2007). To quantify the content of foliar nutrient elements refer to Bao (2000) for the specific determination methods of the following indicators:

1. Total carbon of leaves: refer to the potassium dichromate—sulfuric acid oxidation method, oil bath, and elimination cooking;
2. Total nitrogen of leaves: refer to the semi-trace Kjeldahl method using concentrated H_2_SO_4_ and desiccated leaves;
3. Total phosphorus in leaves: desiccated leaves in H_2_SO_4_-H_2_O_2_ according to the mo-Sb resistance colorimetric method;
4. Total potassium of leaves: the flame photometer method with H_2_SO_4_–H_2_O_2_ elimination.

Moreover, the main foliar nutrients, including amino acid, soluble sugar, and soluble protein were quantitatively determined:

1. Amino acids (spectrophotometric method): 0.02 N hydrochloric acid extraction, amino acid automatic analyzer for determination;
2. Soluble sugar (micromethod): spectrophotometer (wavelength 620 nm) was used for determination by referring to the anthrone-sulfuric acid method;
3. Soluble protein (spectrophotometric method): spectrophotometer (wavelength 595 nm) was used to measure by referring to the coomath bright blue method.

### Survey of population abundances and community diversity of insects

Insect surveys were conducted seven times each year, the first of which was on July 25 and continued 10 days apart. In each instance, ten corn plants with uniform growth were randomly selected in each plot for all of the monoculture and intercropping treatments with normal fertilizer and reduced fertilizer by using the five-point sampling method, respectively. All insect pests and natural enemies on the selected plants were investigated and their species and numbers were counted. In the present study, three insect community indexes [namely the Shannon-Wiener diversity index (*H*), Pielou evenness index (*E*), and Simpson dominant concentration (*D*)] and Species number (*S*) were calculated based on the species and number of sampled insects for every evaluation. Seven results were obtained for each exponent each year. The formulas were following as:

Shannon-Wiener diversity index:


$$H=-\overset{S}{\underset{i=1}{\sum }}Pi\times \ln\left(Pi\right)Pi=Ni/N$$

Pielou evenness index:


$$E=H/H_{\text{max}}H_{\text{max}}=\ln\;S$$

Simpson dominance index:


$$D=\overset{S}{\underset{i=1}{\sum }}\left(Pi\right)2Pi=Ni/N$$

*P_i_*: relative abundance of insect species *i*; *N_i_*: number of individuals for species *i*; *N*: the total number of individuals of all species in the community; *S*: the number of species in the community; *H_max_*: Maximum species diversity index.

The population dynamics of the Asian corn borer on both intercrops and monoculture crops were surveyed during the growing seasons, and the surveys were done at the same time as the insect community surveys.

### Data analysis

All data were analyzed with SPSS 20.0 (IBM Inc., Armonk, NY, United States). Three-way repeated-measured ANOVAs were used to analyze the effects of intercropping type (T; corn–soybean/peanut/millet intercropping), planting pattern (P; three intercropping planting patterns of corn with soybean/peanut/millet, and corn monocultures), fertilizer level (F; normal and reduced fertilizer levels), and their interactions on community diversity of insects on the corn plants in 2018 and 2019. Seven evaluations were used as repeated data. Moreover, three-way ANOVAs were used to analyze the effects of intercropping type (T), planting pattern (P), fertilizer level (F), and their interaction on the population dynamic of Asian corn borer, the foliar contents and elements of nutrients, JA, and SA of corn plants in 2018 and 2019. The least significant difference (LSD) test was used to analyze the significant differences among treatments at *p* < 0.05.

## Results

### Community of insects, population dynamics of key herbivorous insects, and species number of natural enemy insects on corn plants intercropping with soybean, peanut, and millet under normal and reduced fertilizer

A three-way repeated-measured ANOVAs on the four measured indices (namely *S*, *H*, *E*, and *D*) of insects indicated that under the three intercropping types of corn–soybean, corn–peanut, and corn–millet, the four indices of insect community diversity were significantly different in the different corn planting patterns (*p* < 0.001) in 2018 and 2019 (Table 2). In addition, intercropping type (*p* = 0.010) and fertilizer (*p* = 0.033) also had significant effects on species numbers (*S*) in 2018. The interaction of the three factors had a significant effect on *S* (*p* = 0.046) in 2018, and the interaction of fertilizer and intercropping type had a significant effect on *E* (*p* = 0.029) in 2019 (Table 2).

**Table 2:** Three-way repeated-measured analysis of variance (ANOVA) on the effects of intercropping type (T; corn–soybean/peanut/millet intercropping), planting patterns (P; corn monoculture and three planting patterns of corn intercropping with soybean/peanut/millet, respectively), fertilizer level (F; normal vs. reduced), and their bi-/tri-interactions on the community diversity indexes of insects, number of natural enemy insect species on corn plants, and three-way ANOVA on the population dynamics of Asian corn borer per 30 corn plants in 2018 and 2019 (*F/p* value).

Year	Source of variation	*S*	*H*	*E*	*D*	Species number of natural enemy insect	Population dynamics of Asian corn borer
2018	Intercropping types (T)	4.7/0.010^*^	2.7/0.067	0.6/0.543	1.6/0.212	0.2/0.794	11.6/<0.001^***^
	Planting pattern (P)	5.8/<0.001^***^	18.7/<0.001^***^	7.3/<0.001^***^	52.0/<0.001^***^	3.0/0.049^*^	3.5/<0.001^***^
	Fertilizer level (F)	5.1/0.033^*^	1.1/0.293	0.1/0.821	0.1/0.773	<0.1/0.871	<0.1/0.928
	F × P	2.1/0.075	1.0/0.434	0. 5/0.885	1.0/0.378	1.6/0.205	0.2/0.996
	F × T	1.1/0.342	2.4/0.092	0.3/0.707	1.0/0.362	0.3/0.754	0.2/0.817
	P × T	0.6/0.698	0.3/0.867	0.9/0.442	0.3/0.865	0.8/0.556	0.1/0.992
	F × P × T	2.4/0.046^*^	0.3/0.849	0.6/0.692	0.4/0.787	0.4/0.842	0. 3/0.895
2019	Intercropping types (T)	1.2/0.287	1.6/0.207	2.0/0.078	2. 2/0.117	1.3/0.260	9.4/<0.001^***^
	Planting pattern (P)	18.4/<0.001^***^	66.9/<0.001^***^	28.8/<0.001^***^	61.7/<0.001^***^	0.1/0.906	3.2/0.002^**^
	Fertilizer level (F)	1.4/0.243	6.6/0.010^*^	<0.1/0.863	<0.1/0.949	0.4/0.670	3.1/0.082
	F × P	1.1/0.352	0.7/0.533	0.4/0.764	0.1/0.946	1.4/0.228	0.3/0.973
	F × T	2.1/0.128	2.0/0.130	2.5/0.029^*^	0.1/0.925	0.4/0.667	0.6/0.574
	P × T	0.3/0.850	1.6/0.177	0.8/0.558	0.8/0.519	1.4/0.246	0.4/0.794
	F × P × T	0.2/0.928	0.4/0.805	<0.1/0.998	0.1/0.973	0.4/0.811	0.2/0.937

* *p* < 0.05;

** *p* < 0.01; and

*** *p* < 0.001.

*S*-Species number of insect; *H*-Shannon-Wiener diversity index; *E*-Pielou evenness index; *D*-Simpson dominance index. The same as the following tables.

Analysis of the data from both years showed that intercropping type and planting pattern had significant effects on the number of Asian corn borer moths (*Ostrinia furnacalis*, Guenée) on corn plants (*p* < 0.05). Conversely, fertilizer level (*p* ≥ 0.05) had no significant effects on the numbers (Table 2).

According to the analysis of two-year data, intercropping type and fertilizer level had no significant effect on the number of natural enemy insect species of corn in both 2018 and 2019 (*p* > 0.05, Table 2). The planting pattern, however, had a significant effect on this (*p* = 0.049) in 2018 and no significant effect in 2019 (*p* = 0.906; Table 2).

#### Community diversity of insects

**Figure 1:**
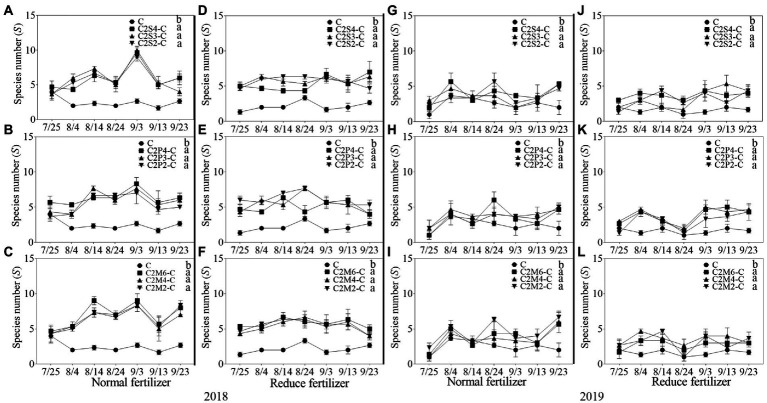
The average species number (*S*) each survey of insect communities on corn plants intercropping with soybean, peanut, and millet under different planting patterns at normal and reduced fertilizer. **A-C** represent corn-soybean intercropping, corn-peanut intercropping and corn-millet intercropping under normal fertilizer in 2018, respectively. **D-F** represent corn- soybean intercropping, corn-peanut intercropping and corn-millet intercropping under fertilizer reduction in 2018, respectively. **G-I** represents corn-soybean intercropping, corn-peanut intercropping and corn-millet intercropping under normal fertilizer in 2019, respectively. **J-L** represent corn-soybean intercropping, corn- peanut intercropping and corn-millet intercropping under fertilizer reduction in 2019, respectively. C2S2-C, C2S3-C, C2S4-C: corn plants in the planting patterns of 2 corn rows intercropping with 2, 3, 4 rows of soybean; C2P2-C, C2P3-C, C2P4-C: corn plants in the planting patterns of 2 corn rows intercropping with 2, 3, 4 rows of peanut; C2M2-C, C2M4-C, C2M6-C: corn plants in the planting patterns of 2 corn rows intercropping with 2, 4, 6 rows of millet. Different lowercase represents a significantly different average of the 7 evaluations among different planting patterns at the same fertilizer level and the same intercropping type by the LSD test at *p* < 0.05, respectively.

**Figure 2:**
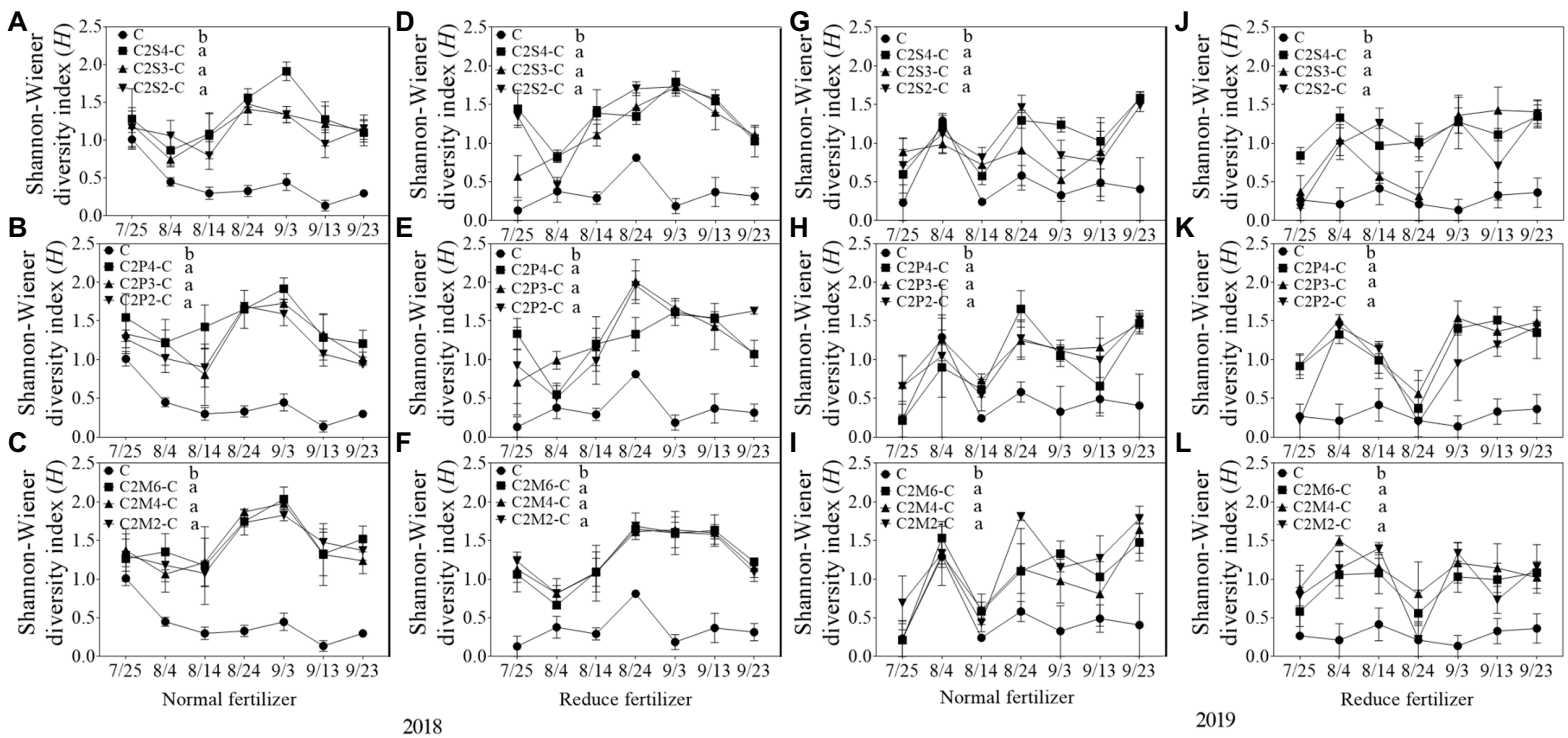
Shannon–Wiener diversity index (H) of insect communities on corn plants intercropping with soybean, peanut, and millet under different planting patterns at normal and reduced fertilizer. **A-C** represent corn-soybean intercropping, corn-peanut intercropping and corn-millet intercropping under normal fertilizer in 2018, respectively. **D-F** represent corn- soybean intercropping, corn-peanut intercropping and corn-millet intercropping under fertilizer reduction in 2018, respectively. **G-I** represents corn-soybean intercropping, corn-peanut intercropping and corn-millet intercropping under normal fertilizer in 2019, respectively. **J-L** represent corn-soybean intercropping, corn- peanut intercropping and corn-millet intercropping under fertilizer reduction in 2019, respectively. C2S2-C, C2S3-C, C2S4-C: corn plants in the planting patterns of 2 corn rows intercropping with 2, 3, 4 rows of soybean; C2P2-C, C2P3-C, C2P4-C: corn plants in the planting patterns of 2 corn rows intercropping with 2, 3, 4 rows of peanut; C2M2-C, C2M4-C, C2M6-C: corn plants in the planting patterns of 2 corn rows intercropping with 2, 4, 6 rows of millet. Different lowercase represents a significantly different average of the 7 evaluations among different planting patterns at the same fertilizer level and the same intercropping type by the LSD test at *p* < 0.05, respectively.

**Figure 3:**
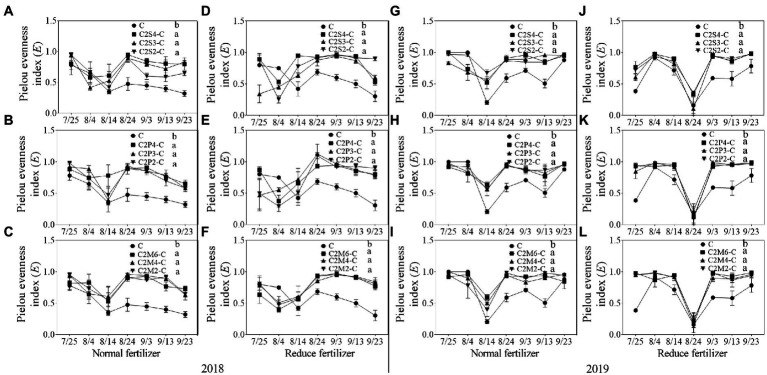
Pielou evenness index (E) of insect communities on corn plants intercropping with soybean, peanut, and millet under different planting patterns at normal and reduced fertilizer. **A-C** represent corn-soybean intercropping, corn-peanut intercropping and corn-millet intercropping under normal fertilizer in 2018, respectively. **D-F** represent corn- soybean intercropping, corn-peanut intercropping and corn-millet intercropping under fertilizer reduction in 2018, respectively. **G-I** represents corn-soybean intercropping, corn-peanut intercropping and corn-millet intercropping under normal fertilizer in 2019, respectively. **J-L** represent corn-soybean intercropping, corn- peanut intercropping and corn-millet intercropping under fertilizer reduction in 2019, respectively. C2S2-C, C2S3-C, C2S4-C: corn plants in the planting patterns of 2 corn rows intercropping with 2, 3, 4 rows of soybean; C2P2-C, C2P3-C, C2P4-C: corn plants in the planting patterns of 2 corn rows intercropping with 2, 3, 4 rows of peanut; C2M2-C, C2M4-C, C2M6-C: corn plants in the planting patterns of 2 corn rows intercropping with 2, 4, 6 rows of millet. Different lowercase represents a significantly different average of the 7 evaluations among different planting patterns at the same fertilizer level and the same intercropping type by the LSD test at *p* < 0.05, respectively.

**Figure 4:**
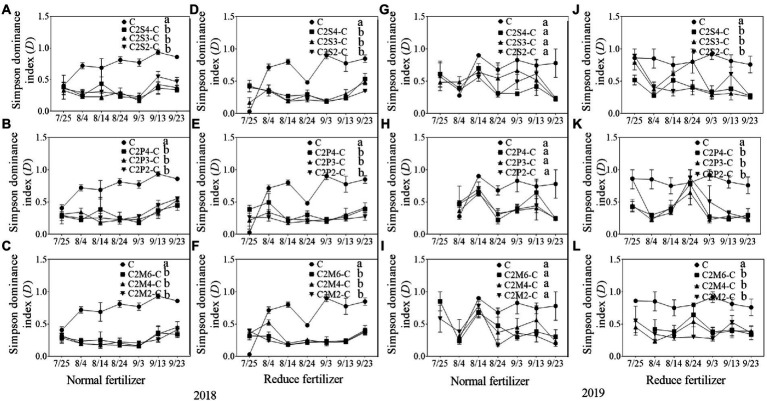
Simpson dominance index (D) of insect communities on corn plants intercropping with soybean, peanut, and millet under different planting patterns at normal and reduced fertilizer. **A-C** represent corn-soybean intercropping, corn-peanut intercropping and corn-millet intercropping under normal fertilizer in 2018, respectively. **D-F** represent corn- soybean intercropping, corn-peanut intercropping and corn-millet intercropping under fertilizer reduction in 2018, respectively. **G-I** represents corn-soybean intercropping, corn-peanut intercropping and corn-millet intercropping under normal fertilizer in 2019, respectively. **J-L** represent corn-soybean intercropping, corn- peanut intercropping and corn-millet intercropping under fertilizer reduction in 2019, respectively. C2S2-C, C2S3-C, C2S4-C: corn plants in the planting patterns of 2 corn rows intercropping with 2, 3, 4 rows of soybean; C2P2-C, C2P3-C, C2P4-C: corn plants in the planting patterns of 2 corn rows intercropping with 2, 3, 4 rows of peanut; C2M2-C, C2M4-C, C2M6-C: corn plants in the planting patterns of 2 corn rows intercropping with 2, 4, 6 rows of millet. Different lowercase represents a significantly different average of the 7 evaluations among different planting patterns at the same fertilizer level and the same intercropping type by the LSD test at *p* < 0.05, respectively.

In 2018, compared with corn monoculture, *S* of different intercropping types under normal fertilizer significantly increased by 2.9–4.5 (Figures 1A–C); *H* significantly increased by 0.7–1.1 (Figures 2A–C); *E* significantly increased by 0.2–0.3 (Figures 3A–C); and *D* significantly decreased by 0.4–0.5 (Figures 4A–C). Under reduced fertilizer, *S* significantly increased by 2.9–4.0 (Figures 1D–F); *H* significantly increased by 0.8–1.0 (Figures 2D–F); the *E* increased by 0.1–0.2 significantly (Figures 3D–F); and *D* significantly decreased by 0.3–0.4 (Figures 4D–F).

In 2019, compared with corn monoculture, *S* of different intercropping types under normal fertilizer significantly increased by 1.0–2.0 (Figures 1G–I); *H* significantly increased by 0.4–0.7 (Figures 2G–I); *E* significantly increased by 0.1–0.2 (Figures 3G–I); and *D* significantly decreased by 0.2–0.3 (Figures 4G–I). Under reduced fertilizer, *S* significantly increased by 1.1–2.2 (Figures 1J–L); *H* significantly increased by 0.6–0.9 (Figures 2J–L); *E* significantly increased by 0.2–0.3 (Figures 3J–L); and *D* significantly decreased by 0.3–0.5 (Figures 4J–L).

#### Asian corn borer

**Figure 5:**
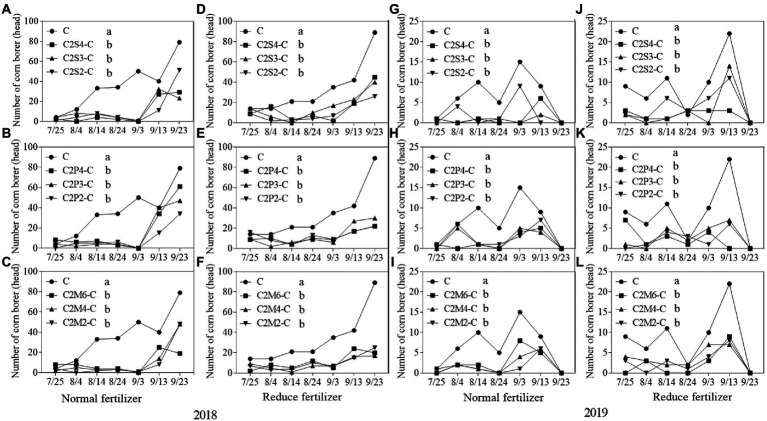
Population dynamics of key herbivorous insect, Asian corn borers, that fed on per 30 corn plants intercropping with soybean, peanut, and millet under different planting patterns at normal and reduced fertilizer per 30 corn plants. **A-C** represent corn-soybean intercropping, corn-peanut intercropping and corn-millet intercropping under normal fertilizer in 2018, respectively. **D-F** represent corn- soybean intercropping, corn-peanut intercropping and corn-millet intercropping under fertilizer reduction in 2018, respectively. **G-I** represents corn-soybean intercropping, corn-peanut intercropping and corn-millet intercropping under normal fertilizer in 2019, respectively. **J-L** represent corn-soybean intercropping, corn- peanut intercropping and corn-millet intercropping under fertilizer reduction in 2019, respectively. C2S2-C, C2S3-C, C2S4-C: corn plants in the planting patterns of 2 corn rows intercropping with 2, 3, 4 rows of soybean; C2P2-C, C2P3-C, C2P4-C: corn plants in the planting patterns of 2 corn rows intercropping with 2, 3, 4 rows of peanut; C2M2-C, C2M4-C, C2M6-C: corn plants in the planting patterns of 2 corn rows intercropping with 2, 4, 6 rows of millet. Different lowercase represents a significantly different average of the 7 evaluations among different planting patterns at the same fertilizer level and the same intercropping type by the LSD test at *p* < 0.05, respectively.

Compared with monoculture corn plants, under normal fertilizer, the number of Asian corn borers on intercropping corn plants significantly decreased by 52.8–74.6% in all the planting patterns (Figures 5A–C) in 2018. Furthermore, the number of Asian corn borers on intercropping corn plants with reduced fertilizer significantly decreased by 53.4–73.7% in all the planting patterns (Figures 5D–F) in 2018. In 2019, compared to corn monoculture, under normal fertilizer, the number of Asian corn borers on intercropping corn plants significantly decreased by 60.9–91.3% (Figures 5G–I). Similarly, the number of Asian corn borers on intercropping corn plants with reduced fertilizer significantly decreased by 40.8–71.4% (Figures 5J–L) in 2019.

There was a significant difference between intercropping and monoculture. The average of each survey under different fertilizers and planting patterns was calculated separately. Compared with monoculture, the number of Asian corn borers on intercropping plants (corn–soybean/ corn–peanut/corn–millet) decreased by 9.1–17.0 heads per 30 corn plants under the normal fertilizer level per 30 corn plants in 2018 (Figures 5A–C). Furthermore, compared with monoculture, the number of Asian corn borers on intercropping plants under reduced fertilizer decreased by 8.9–15.7 heads per 30 corn plants in 2018 (Figures 5D–F). In 2019, compared with monoculture, the number of Asian corn borers in intercropping plants decreased by 4.0–6.0 heads per 30 corn plants under normal fertilizer (Figures 5G–I). Finally, compared with monoculture, the number of Asian corn borers on intercropping plants under reduced fertilizer decreased by 2.9–5.0 heads per 30 corn plants in 2019 (Figures 5J–L).

#### Species number of natural enemy insects

**Figure 6:**
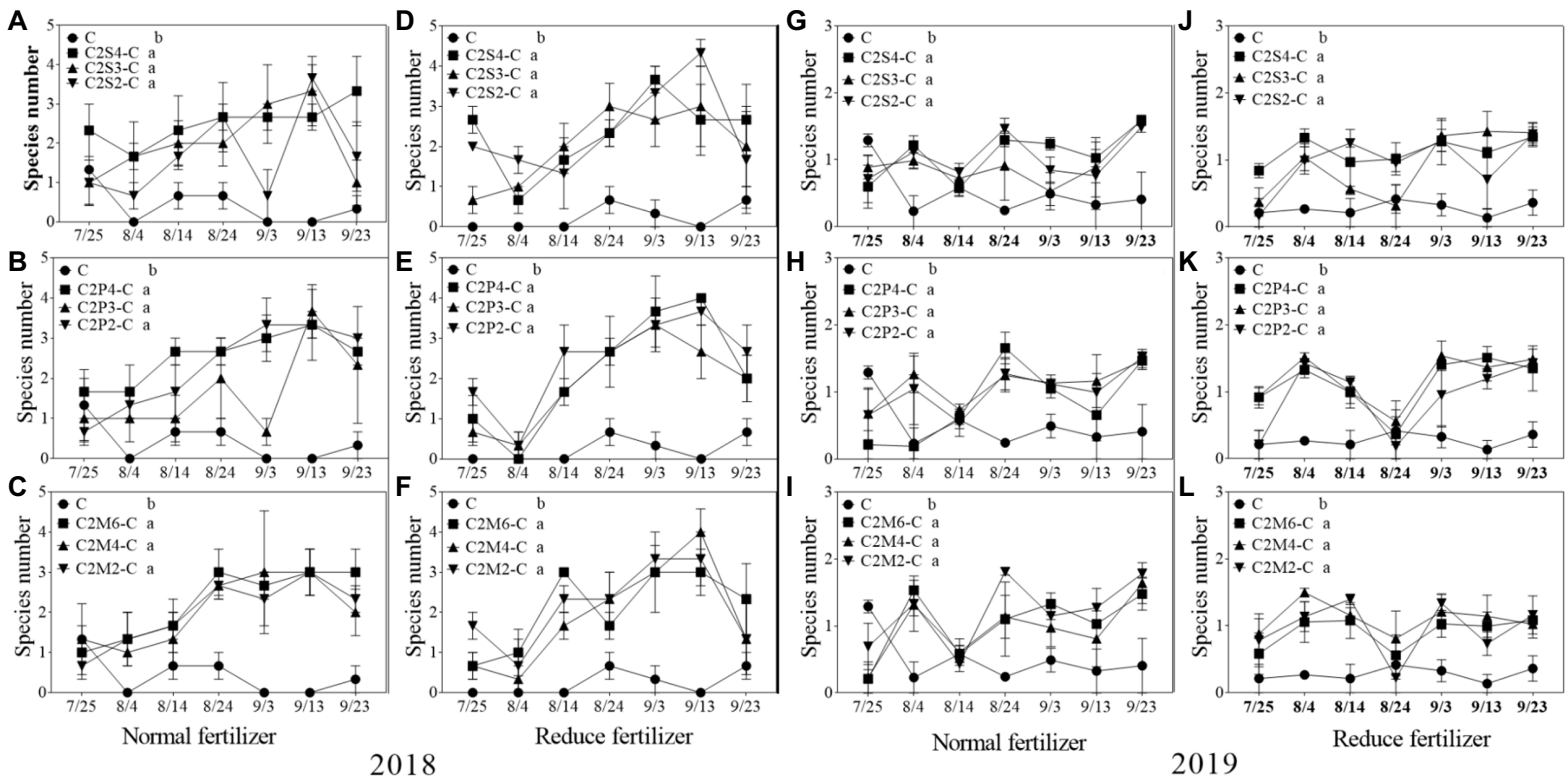
Species number (*S*) of natural enemy insect on corn plants intercropping with soybean, peanut and millet under different planting patterns at normal and reduced fertilizer. **A-C** represent corn-soybean intercropping, corn-peanut intercropping and corn-millet intercropping under normal fertilizer in 2018, respectively. **D-F** represent corn- soybean intercropping, corn-peanut intercropping and corn-millet intercropping under fertilizer reduction in 2018, respectively. **G-I** represents corn-soybean intercropping, corn-peanut intercropping and corn-millet intercropping under normal fertilizer in 2019, respectively. **J-L** represent corn-soybean intercropping, corn- peanut intercropping and corn-millet intercropping under fertilizer reduction in 2019, respectively. C2S2-C, C2S3-C, C2S4-C: corn plants in the planting patterns of 2 corn rows intercropping with 2, 3, 4 rows of soybean; C2P2-C, C2P3-C, C2P4-C: corn plants in the planting patterns of 2 corn rows intercropping with 2, 3, 4 rows of peanut; C2M2-C, C2M4-C, C2M6-C: corn plants in the planting patterns of 2 corn rows intercropping with 2, 4, 6 rows of millet. Different lowercase represents a significantly different average of the 7 evaluations among different planting patterns at the same fertilizer level and the same intercropping type by the LSD test at *p* < 0.05, respectively.

Under normal fertilizer, the average number of natural enemy species on intercropping corn plants was significantly increased by 1.2–2.1 species compared with monoculture in 2018; under the reduced fertilizer, the number significantly increased by 1.7–2.2 species. In 2019, compared with monoculture, the number of natural enemy insect species on intercropping corn significantly increased by 0.3–0.7 species under normal fertilizer; and significantly increased by 0.6–0.9 species under the reduced fertilizer in 2019 (Figure 6).

### Nutrient composition of corn leaves

Intercropping type (T), planting pattern (P), and fertilizer level (F) had significant effects on the total amino acids, soluble proteins, and soluble sugars in corn leaves (*p* < 0.05; Table 3). The three factors had the same influence trend on the contents of the three nutrients.

**Table 3:** Three-way ANOVAs about the effects of intercropping type (T; corn–soybean/peanut/millet intercropping), planting patterns (P; one corn monoculture and three planting patterns of corn intercropping with soybean/peanut/millet, respectively), fertilizer level (F; normal vs. reduced) and their bi-/tri-interactions on foliar contents of nutrients and plant hormones in 2018 and 2019 (*F*/*p* value).

Year	Source of variation	Amino acid	Soluble protein	Soluble sugar	Jasmonic acid	Salicylic acid
2018	Intercropping type (T)	26.9/<0.001^***^	72.4/<0.001^***^	108.7/<0.001^***^	43.3/<0.001^***^	185.7/<0.001^***^
	Planting pattern (P)	4.5/0.017^*^	8.4/<0.001^***^	3.8/0.001^***^	11.1/<0.001^***^	9.3/<0.001^***^
	Fertilizer (F)	524.4/<0.001^***^	144.8/<0.001^***^	294.9/<0.001^***^	18.4/<0.001^***^	430.6/<0.001^***^
	F × P	2.1/0.132	0.1/0.889	3.1/0.058	0.4/0.662	0.1/0.899
	F × T	3.3/0.046^*^	1.6/0.220	1.8/0.174	0.2/0.840	3.3/0.046^*^
	P × T	0.6/0.662	2.2/0.087	1.9/0.136	1.1/0.391	8.8/<0.001^***^
	F × P × T	<0.05/0.996	0.9/0.501	1.3/0.292	0.4/0.841	28.5/<0.001^***^
2019	Intercropping type (T)	52.3/<0.001^***^	178.1/<0.001^***^	152.1/<0.001^***^	71.6/<0.001^***^	136.6/<0.001^***^
	Planting pattern (P)	4.5/<0.001^***^	22.7/<0.001^***^	5.5/0.008^***^	7.9/0.001^***^	12.7/<0.001^***^
	Fertilizer (F)	142.6/<0.001^***^	132.6/<0.001^***^	225.8/<0.001^***^	50.6/<0.001^***^	126.1/<0.001^***^
	F × P	0.7/0.498	1.3/0.283	0.1/0.841	0.2/0.820	0.1/0.874
	F × T	2.7/0.082	0.2/0.788	6.2/0.005^**^	1.6/0.207	0.1/0.915
	P × T	0.7/0.585	1.3/0.297	1.6/0.200	0.2/0.924	2.1/0.097
	F × P × T	1.7/0.174	1.6/0.201	1.8/0.157	0.4/0.803	1.9/0.126

* *p* < 0.05;

** *p* < 0.01; and

*** *p* < 0.001.

The same as the following tables.

Compared with monoculture, the total amino acid content in corn leaves increased by 10.4–14.6% (corn–soybean), 8.6–12.5% (corn–peanut), and 4.3–10.7% (corn–millet) under normal fertilizer in 2018 (Table 4); and increased by 20.7–26.5% (corn–soybean), 17.2–19.2% (corn–peanut) and 4.0–10.8% (corn–millet) under reduced fertilizer (Table 4). Compared with monoculture, the total amino acid content in corn leaves increased by 6.3–7.7% (corn–soybean), 3.9–6.0% (corn–peanut) and 0.9-7.7% (corn-millet) under normal fertilizer in 2019 (Table 4); and increased by 7.9–10.7% (corn–soybean), 3.3–6.5% (corn–peanut), and 1.8–3.6% (corn–millet) under reduced fertilizer (Table 4). Under the same planting pattern, compared with normal fertilizer, reduced fertilizer significantly decreased the contents of total amino acids in corn leaves by 14.8–25.4% in each planting pattern in 2018 (Table 4); and significantly decreased the contents of total amino acids in corn leaves by 2.8–8.2% in 2019 (Table 4).

**Table 4:** Effects of different intercropping planting patterns and fertilizer levels on the contents of nutrients (AA, SP and SS) and secondary metabolites (JA and SA) in corn leaves in 2018 and 2019.

Year	Intercropping planting pattern	Amino acid		Soluble protein		Soluble sugar		Jasmonic acid		Salicylic acid	
		N	R	N	R	N	R	N	R	N	R
2018	C	890.8Ab	669.3Bc	8.1Acd	7.3Bc	3.2Acd	2.9 Bd	8.5Bcd	9.7Aab	68.6Ba	72.0Aa
	C2S2-C	983.6Aab	807.7Bab	8.8Abc	7.9Babc	4.1Aa	3.1Bbc	10.7Abc	13.6Aab	45.0Bcd	51.4Ac
	C2S3-C	992.9Aa	846.4Ba	9.0Aab	8.3Bab	3.8Aab	3.6Bab	12.0Aab	14.9Aa	41.7 Bd	60.0Ab
	C2S4-C	1020.7Aa	830.1Ba	9.6Aa	8.5Ba	4.2Aa	3.5Ba	13.8Aa	14.6Aab	30.1Be	57.2Abc
	C2P2-C	982.2Aab	784.3Bab	8.3Abcd	7.4Bbc	3.5Abcd	3.0Bcd	9.2Abcd	11.4 Aab	44.7Bcd	60.7Ab
	C2P3-C	967.4Aab	795.5Bab	8.5Abcd	7.3Bc	3.8Abc	3.0Bcd	10.3Abcd	12.4Aab	41.8 Bd	58.6Abc
	C2P4-C	1001.7Aa	798.1Bab	8.5Abcd	7.6Bc	3.4Abcd	3.1Bcd	10.8Bbc	12.4Aab	45.1Bcd	60.5Ab
	C2M2-C	933.0Aab	695.8Bc	7.9Acd	7.3Bc	3.1Acd	2.7 Bd	8.2Bcd	9.5Aab	48.7Bc	72.8Aa
	C2M4-C	928.8Aab	734.3Bbc	8.1Acd	7.3Bc	3.2Ad	2.7 Bd	7.5 Bd	9.3Ab	59.9Bb	70.6Aa
	C2M6-C	986.0Aab	741.3Bbc	8.1Acd	7.4Bc	3.3Acd	2.9 Bd	7.8 Bd	9.5Ab	57.2Bb	62.6Ab
2019	C	919.0Ae	866.1 Bd	8.1Ae	7.5Be	3.3Ad	3.0Bc	9.3Bbc	10.7Acd	62.2Ba	66.5Aa
	C2S2-C	976.9Aabc	934.8Bab	9.2Abc	8.9Ba	4.0Aab	3.2Bab	10.7Aab	13.3Aabc	41.2Be	50.0Acd
	C2S3-C	989.7Aa	944.0Ba	9.4Aab	8.9Ba	3.9Aab	3.6Ba	11.9Aa	13.7Aba	41.3Be	50.3Acd
	C2S4-C	986.6Aab	958.8Ba	9.7Aa	9.1Ba	4.2Aa	3.6Ba	12.4Aa	14.4Aa	39.7Be	48.0Ad
	C2P2-C	973.9Aabcd	894.5Bcd	8.7Acd	8.4Bbc	3.3Acd	3.0Bbc	9.6Abc	10.8Acd	50.0Bcd	62.3Aab
	C2P3-C	955.2Aabcde	903.0Bbc	8.9Acd	8.6Bbc	3.5Acd	3.0Bc	9.9Abc	11.1Abcd	48.6Bcd	57.1Abc
	C2P4-C	964.6Aabcd	922.6Babc	9.0Ac	8.7Bab	3.5Abc	3.0Bbc	10.6Abc	11.6Aabcd	45.1Bde	52.3Acd
	C2M2-C	937.8Ade	892.9Bbcd	8.2Ae	7.8 Bde	3.3Ad	2.8Bc	8.2Bc	9.7Ad	58.6Bab	64.7Aab
	C2M4-C	927.4Acde	881.7Bcd	8.5Ade	8.0Bcde	3.1Ad	3.0Bc	8.7Bbc	10.2Ad	55.0Babc	62.8Aab
	C2M6-C	943.9Abcde	897.1Bcd	8.7Acd	8.2Bbcd	3.2Ad	3.0Bc	8.7Bbc	11.1Abcd	51.5Bbcd	64.0Aab

Soybean (S), peanut (P), millet (M), and corn (C) respectively. C2S2, C2S3, C2S4: intercropping of 2 corn rows with 2, 3, 4 rows of soybean respectively. C2P2, C2P3, C2P4: intercropping of 2 corn rows with 2, 3, 4 rows of peanut respectively. C2M2, C2M4, C2M6: intercropping of 2 corn rows with 2, 4, 6 rows of millet, respectively. AA: amino acid; SP: soluble protein; SS: soluble sugar; JA: jasmonic acid; SA: salicylic acid. Different uppercase letters represent significant differences between fertilizer levels in the same year, and different lowercase letters represent significant differences among different intercropping planting patterns under the same fertilizer level in the same year (*p* < 0.05), respectively. N: normal fertilizer level; R: reduced fertilizer level. The table below is the same as this.

Compared with monoculture, the soluble protein content in corn leaves increased by 9.0–19.1% under the normal fertilizer and significantly increased by 7.9–16.6% under reduced fertilizer for corn–soybean in 2018 (Table 4). Furthermore, compared with monoculture, the soluble protein content in corn leaves significantly increased by 9.0–19.9% (corn–soybean) and 7.5–11.1% (corn–peanut) under normal fertilizer (Table 4); and significantly increased by 17.4–20.6% (corn–soybean) and 10.9–14.7% (corn–peanut) under the reduced fertilizer in 2019 (Table 4). There was no significant difference among corn–millet intercropping with monoculture. Under the same cropping pattern, compared with normal fertilizer, the reduced fertilizer significantly reduced the soluble protein content of corn leaves by 7.6–13.8% in 2018 (Table 4), and reduced fertilizer significantly decreased the soluble protein content by 2.6–7.1% in 2019 (Table 4).

Compared with monoculture, the soluble sugar content in corn leaves under the normal fertilizer significantly increased by 16.2–22.3% (corn–soybean) and increased 6.1–8.5% (corn–peanut) in 2018 (Table 4). Meanwhile, the soluble sugar content in corn leaves of corn–soybean intercropping was significantly higher than in corn–peanut intercropping. Under the reduced fertilizer, it increased by 14.3–27.0% (corn–soybean) and 8.0–9.9% (corn–peanut) in 2018 (Table 4). Similarly, the soluble sugar content in corn leaves of corn–soybean intercropping was higher than in corn–peanut intercropping under reduced fertilizer. In 2019, compared with monoculture, the soluble sugar content in corn leaves under normal fertilizer significantly increased by 19.8–22.1% (corn–soybean) and increased 2.0–11.1% (corn–peanut; Table 4); meanwhile, the soluble sugar content in corn leaves of corn–soybean intercropping was higher than corn–peanut intercropping. Under the reduced fertilizer, it significantly increased by 10.4–18.4% (corn–soybean) in 2019 (Table 4).

In this part, the difference between individual planting patterns and monoculture may not reach a significant level, such as for C2S2-C and C. However, according to the data, the nutrient content of corn leaves under intercropping did increase.

### JA and SA contents

The data analysis for two consecutive years showed that intercropping type (T), planting pattern (P), and fertilizer (F) had significant effects on the contents of JA and SA in corn leaves in 2018 and 2019 (*p* < 0.001; Table 3). In 2018, data analysis showed that the interaction between/among different factors had a significant impact on SA content, F × T (*p* = 0.046; Table 3), P × T (*p* < 0.001; Table 3), and F × P × T (*p* < 0.001; Table 3). Compared with monoculture, the JA content in leaves of intercropping corn under normal fertilizer increased by 25.8–62.8% (corn–soybean) and 8.0–26.7% (corn–peanut); and increased by 15.2–34.3% (corn–soybean) and 3.6–14.5% (corn–peanut) under reduced fertilizer in 2018 (Table 4). In 2018, the JA content in corn leaves from corn–soybean intercropping was higher than in corn–peanut intercropping. Furthermore, the content from corn–peanut intercropping was significantly higher than from corn–millet intercropping. There was no significant difference between corn–millet intercropping and corn monoculture. In 2019, compared with monoculture, JA content under normal fertilizer increased by 40.2–50.6% (corn–soybean) and increased by 24.1–34.6% (corn–soybean) under the reduced fertilizer (Table 4). There was no significant difference between monoculture and other intercropping types. Under the same planting pattern, compared with normal fertilizer level, reduced fertilizer significantly increased the JA content in corn leaves by 5.4–26.9% in all the planting patterns in 2018 (Table 4), and the reduced fertilizer significantly increased the JA content in corn leaves by 9.4–27.2% in all the planting patterns in 2019 (Table 4).

For intercropping types, the SA content in corn leaves of corn monoculture and corn–millet intercropping was significantly higher than in corn–soybean and corn–peanut intercropping in 2018 and 2019. The SA content in corn leaves from corn–peanut intercropping was significantly higher than in corn–soybean intercropping in 2019. Compared with monoculture, the SA content of corn leaves under intercropping with normal fertilizer decreased by 34.4–56.1% (corn–soybean) and 34.2–39.1% (corn–peanut); and decreased by 33.7–36.1% (corn–soybean) and 19.5–27.5% (corn–peanut) with reduced fertilizer in 2018 (Table 4). In 2019, compared with monoculture, the SA content in corn leaves decreased by 16.7–28.6% (corn–soybean) and 15.7–18.6% (corn–peanut) under the normal fertilizer; and decreased by 24.4–27.8% (corn–soybean) and 6.3–21.4% (corn–peanut) under the reduced fertilizer (Table 4). Under the same planting pattern, compared with normal fertilizer, reduced fertilizer significantly increased the SA content in corn leaves by 4.9–89.9% in all the planting patterns in 2018 (Table 4); and reduced fertilizer significantly increased the SA content in corn leaves by 7.0–24.6% in all the planting patterns in 2019 (Table 4).

### Nutrient elements of corn leaves

Analysis of the data from both years showed that intercropping type and fertilizer level had significant effects on total carbon (C), total nitrogen (N), total phosphorus (P), and total potassium (K) in corn leaves (*p* < 0.001, Table 5). Further, the planting pattern had significant effects on the total carbon, total nitrogen, and total phosphorus (*p* < 0.05, Table 5). The interaction between fertilizer level and intercropping type had significant effects on total carbon and total potassium (*p* < 0.05, Table 5) in both years.

**Table 5:** Three-way ANOVAs of the effects of intercropping type (T; corn–soybean/peanut/millet), planting patterns (P; one corn monoculture and three planting patterns of corn intercropping with soybean/peanut/millet), fertilizer level (F; normal vs. reduced), and their bi−/tri-interactions on nutrients element of corn in 2018 and 2019 (*F*/*P* value).

Year	Source of variation	Total C	Total N	Total P	Total K
2018	Intercropping type (T)	141.1/<0.001^***^	124.4/<0.001^***^	339.7/<0.001^***^	42.7/<0.001^***^
	Planting pattern (P)	8.5/<0.001^***^	9.3/<0.001^***^	8.1/0.001^**^	2.8/0.072
	Fertilizer (F)	312. 9/<0.001^***^	198.9/<0.001^***^	152.6/<0.001^***^	125. 4/<0.001^***^
	F × P	0.6/0.541	0.1/0.948	10.0/<0.001^***^	0.1/0.949
	F × T	3.4/0.044^*^	0.2/0.803	4.9/0.013^*^	5.0/0.012^*^
	P × T	2.3/0.076	1.8/0.144	10.8/<0.001^***^	5.5/0.001^**^
	F × P × T	0.9/0.468	1.4/0.236	3.5/0.015^*^	0.8/0.537
2019	Intercropping type (T)	112.8/<0.001^***^	74.0/<0.001^***^	64.3/<0.001^***^	62.0/<0.001^***^
	Planting pattern (P)	5.5/0.008^**^	9.2/<0.001^***^	15.3/<0.001^***^	3.1/0.054
	Fertilizer (F)	307.9/<0.001^***^	117.8/<0.001^***^	120.1/<0.001^***^	150.7/<0.001^***^
	F × P	2.6/0.087	0.2/0.856	0.7/0.500	2. 2/0.129
	F × T	5.7/0.007^**^	2.3/0.111	0.3/0.769	5.6/0.007^**^
	P × T	1. 9/0.137	2.0/0.114	0.7/0.570	1.7/0.173
	F × P × T	4.9/0.003^***^	0.2/0.928	0.4/0.777	0.5/0.709

*p < 0.05; **p < 0.01; ***p < 0.001.

The total carbon (C) content of intercropping corn leaves under normal fertilizer, compared with corn monoculture, decreased by 6.2–9.0% (corn–soybean), and 4.2–5.9% (corn–peanut) in 2018. Furthermore, the total carbon content of corn leaves under reduced fertilizer decreased by 8.0–12.9% (corn–soybean), and 5.4–6.5% (corn–peanut) in 2018 (Table 6). In 2019, compared with corn monoculture, the total carbon content of corn leaves decreased by 6.1–7.3% (corn–soybean), and 4.4–5.7% (corn–peanut) under normal fertilizer in 2019; and decreased by 2.1–9.0% (corn–soybean), and 0–2.7% (corn–peanut) under the reduction of fertilizer application (Table 6). There was no significant difference for corn–millet intercropping. Under the same planting pattern, compared with normal fertilizer, reduced fertilizer increased the total carbon content in corn leaves of monoculture and intercropping by 5.4–10.9% in 2018 (Table 6); and significantly increased by 3.7–12.5% in 2019 (Table 6).

**Table 6:** Effects of different intercropping planting patterns and fertilizer levels on the nutrient elements (C, N, P, and K) in corn leaves in 2018 and 2019.

Year	Intercropping pattern	Total C		Total N		Total P		Total K	
		N	R	N	R	N	R	N	R
2018	C	46.1Bab	51.1Aa	1.7Af	1.5Bf	0.9Ad	0.8 Bd	10.1Ba	12.5Aa
	C2S2-C	43.3Bde	47.1Ade	1.9Aabc	1.8Bab	1.3Aab	1.2Ba	8.6Babc	9.7Abcd
	C2S3-C	42.2Bde	44.5Af	2.0Aa	1.9Ba	1.3Aab	1.2Ba	8.7Bbc	9.6Ad
	C2S4-C	42.0Be	44.6Aef	2.0Aab	1.8Bab	1.4Aa	1.2Ba	9.2Bc	10.1Ad
	C2P2-C	44.2Bbcd	48.4Abcd	1.9Acd	1.7Bbcde	1.0Ac	0.9Bcd	9.3Babc	10.0Aab
	C2P3-C	43.9Bcde	47.8Acd	1.9Aabcd	1.7Bbcd	1.2Ab	1.1Bab	9.8Babc	11.4Aabc
	C2P4-C	43.4Bde	48.0Abcd	1.9Abcd	1.8Babc	1.2Ab	1.0Bbc	10.2Babc	11.6Acd
	C2M2-C	47.1Ba	50.3Aabc	1.7Af	1.6Bef	0.9Ad	0.8 Bd	10.0Babc	12.3Aabc
	C2M4-C	46.9Ba	50.5Aab	1.7Aef	1.6Bdef	0.9Ad	0.8 Bd	10.1Ba	12.0Aa
	C2M6-C	45.8Babc	50.1Aabc	1.8Ade	1.6Bcdef	1.0Acd	0.6Be	9.5Bab	11.5Aa
2019	C	46.1Bab	49.3Aabc	1.8Abc	1.5Be	0.9Ac	0.8Bbcd	10.2Bab	12.9Aa
	C2S2-C	42.9Bc	48.3Abc	1.9Aab	1.9Bab	1.1Aab	0.9Babc	8.8Bbc	9.6Acde
	C2S3-C	42.8Bc	48.0Ac	2.0Aa	1.9Bab	1.1Aa	0.9Bab	8.8Bc	9.8Ade
	C2S4-C	43.3Bc	44.9Ad	2.0Aa	1.9Ba	1.2Aa	1.0Ba	9.0Bc	9.9Ae
	C2P2-C	44.1Bbc	49.3Aabc	1.9Aab	1.7Bbcd	1.0Abc	0.8Bbcd	9.7Bbc	10.7Abcde
	C2P3-C	43.5Bc	48.0Ac	1.9Aab	1.8Babcd	1.1Aab	0.9Babc	9.4Bbc	11.6Aabc
	C2P4-C	43.7Bc	48.2Ac	1.9Aab	1.8Babc	1.1Aab	0.9Babc	9.3Babc	11.5Aabcd
	C2M2-C	47.7Ba	50.4Aab	1.7Ac	1.5Be	0.8Ac	0.7 Bd	10.2Babc	11.8Aab
	C2M4-C	47.5Ba	50.1Aabc	1.8Abc	1.6Bde	0.9Abc	0.8Bbcd	10.8Ba	13.1Aa
	C2M6-C	46.9Ba	50.6Aa	1.9Aabc	1.7Bcde	1.0Abc	0.8Bcd	9.9Bab	12.2Aab

Different uppercase letters represent significant different between fertilizer levels under the same intercropping planting pattern in the same year, and different lowercase letters represent significant different among intercropping planting patterns under the same fertilizer level in the same year (*p* < 0.05).

The total nitrogen (N) content of corn leaves under normal fertilizer, compared with corn monoculture, increased by 16.1–18.7% (corn–soybean) and 11.9–13.5% (corn–peanut) in 2018; and increased by 18.8–25.1% (corn–soybean) and 12.6–18.5% (corn–peanut) under reduced fertilizer in 2018 (Table 6). Compared with corn monoculture, the intercropping type increased the total nitrogen content of corn leaves by 7.6–13.0% (corn–soybean) and 7.7–9.0% (corn–peanut) under normal fertilizer in 2019. Furthermore, total nitrogen content increased by 20.7–24.2% (corn–soybean) and 13.4–15.8% (corn–peanut) under the reduced fertilizer in 2019 (Table 6). Under the same planting pattern, compared with normal fertilizer, the reduced fertilizer decreased the total nitrogen content in corn leaves of monoculture and intercropping by 6.1–11.0% in 2018 (Table 6) and decreased by 3.5–13.9% in 2019 (Table 6).

The total phosphorus(P) content in corn leaves from intercropping, compared with corn monoculture, increased by 41.8–48.1% (corn–soybean) and 13.8–33.9% (corn–peanut) under normal fertilizer in 2018. Furthermore, it increased by 43.9–45.9% (corn–soybean) and 5.0–29.6% (corn–peanut) under reduced fertilizer in 2018 (Table 6). Compared with corn monoculture, the total phosphorus content in corn leaves from intercropping increased by 19.1–30.6% (corn–soybean) and 10.0–22.3% (corn–peanut) under normal fertilizer in 2019; and increased by 18.7–28.5% (corn–soybean) and 2.4–17.3% (corn–peanut) under reduced fertilizer (Table 6). There was no significant difference for corn–millet intercropping. Under the same planting pattern, compared with normal fertilizer, reduced fertilizer decreased the total phosphorus content in leaves of monoculture and intercropping corn by 7.9–39.7% in 2018 (Table 6); and decreased by 13.6–20.9% in 2019 (Table 6).

Compared with corn monoculture, the total potassium (K) content in corn leaves from intercropping decreased by 9.4–15.3% (corn–soybean) and 0–8.6% (corn–peanut) under normal fertilizer in 2018, and decreased by 19.0–23.2% (corn–soybean) and 6.9–20.3% (corn–peanut) under reduced fertilizer (Table 6). Compared with corn monoculture, the total potassium content in corn leaves from intercropping decreased by 11.2–13.5% (corn–soybean) and 4.7–8.4% (corn–peanut) under normal fertilizer in 2019. Further, it decreased by 23.5–25.6% (corn–soybean) and 10.0–17.3% (corn–peanut) under reduced fertilizer in 2019 (Table 6). There was no significant difference for corn–millet intercropping types with corn nomoculture. Under the same planting pattern, compared with normal fertilizer, reduced fertilizer increased the total potassium content in leaves of monoculture and intercropping corn by 7.5–23.3% in 2018 (Table 6); and increased by 9.4–27.3% in 2019 (Table 6).

### Correlation analysis between insect community and corn leaf soluble nutrition, nutrient elements, plant hormone

**Figure 7:**
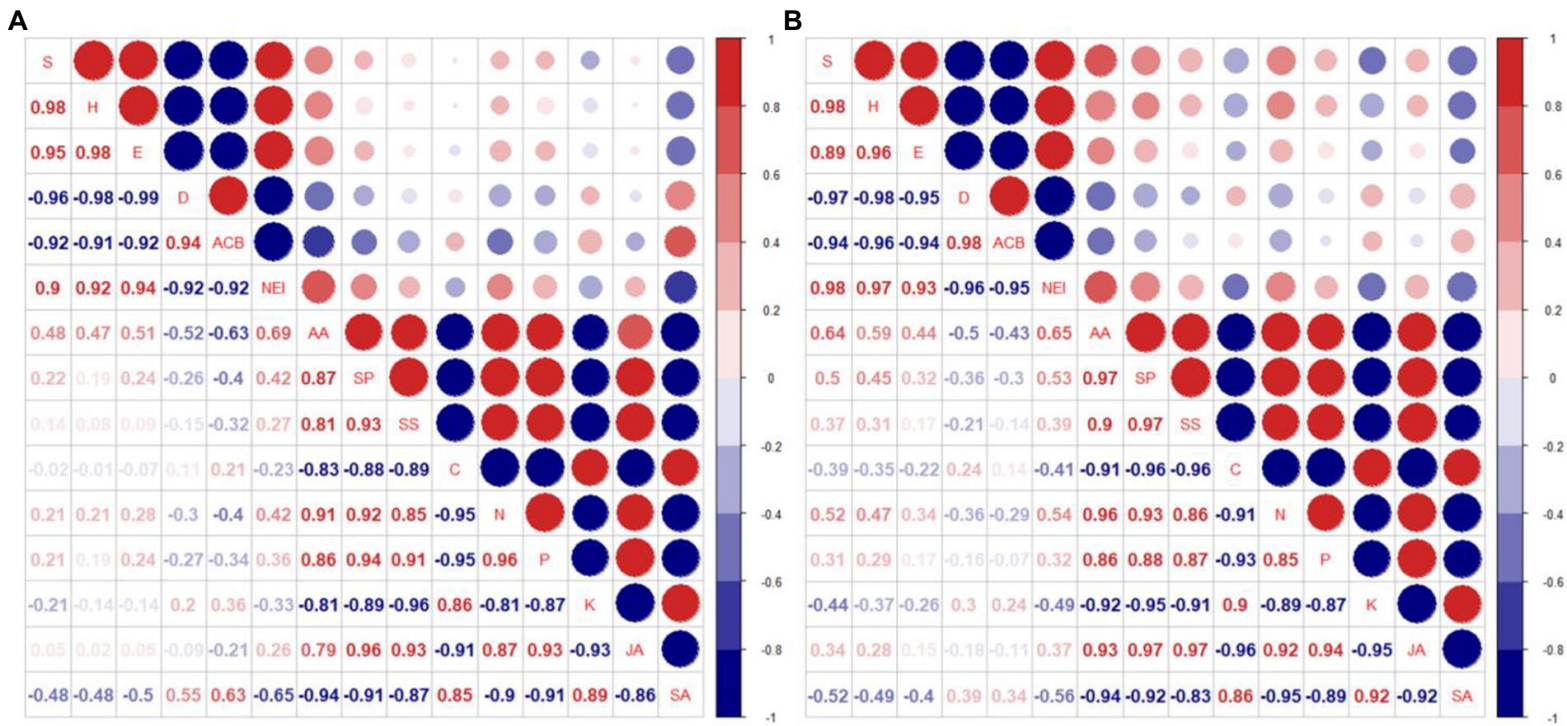
Correlation analysis of insect community index (*S, H, E, D*), the prevalence of Asian corn borers (ACB), number of natural enemy insect species (NEI), corn leaf nutrients (AA, SP, SS), nutrient elements (C, N, P, K), and corn leaf plant hormone (JA, SA) in 2018 and 2019 [**(A)** Normal fertilizer; **(B)** Reduced fertilizer. *S***-**Species number; *H*-Shannon-Wiener diversity index; *E*-Pielou evenness index; *D*-Simpson dominance index; ACB: number of Asian corn borers; NEI: number of natural enemy insect species; AA: amino acid; SP: soluble protein; SS: soluble sugar; C, N, P and K: total carbon, total nitrogen, total phosphorus and total potassium in corn leaves; JA: jasmonic acid; SA: salicylic acid].

It was shown that the correlation trend for normal fertilizer (Figure 7A) and reduced fertilizer (Figure 7B) was consistent.

Nine degrees of freedom, given ∣*r*∣ > 0.602, is significant. The insect diversity index *S*, *H*, and *E* were positively correlated with each other, and with the number of natural enemies. The dominance index *D*, was positively correlated with the prevalence of Asian corn borers. The insect diversity index *S*, *H*, *E*, and the number of natural enemies were negatively correlated with *D* and the prevalence of Asian corn borers. AA, SP, and SS were highly positively correlated with each other. In addition, these 3 nutrients were significantly positively correlated with N, P, and the plant hormone JA in leaves. However, they were significant negative correlated with nutrients C, and K, and the plant hormone, SA. Nutrient elements N and P were significantly positively correlated with JA, and significantly negatively correlated with C, K, and SA. JA was negatively correlated with SA. It can be seen that the increase of two main nutrients, N and P in leaves promoted the synthesis of JA, but decreased the synthesis of SA.

There was a significant negative correlation between AA and the prevalence of Asian corn borer (*r* = −0.63), and a significant positive correlation between AA and the number of natural enemy species (*r* = 0.69) at normal fertilizer (Figure 7A). There was a significant positive correlation between SA and the prevalence of Asian corn borer (*r* = 0.63), and a significant negative correlation between SA and the number of natural enemy species (*r* = −0.65) at normal fertilizer (Figure 7A). There was a significant positive correlation between AA and insect diversity index (*S*) and the number of natural enemy species (*r* = 0.64 and 0.65, respectively) at reduced fertilizer (Figure 7B). However, the other physiological indicators were not significantly correlated with insect community diversity, dominance index (*D*), and the prevalence of Asian corn borers under normal or reduced fertilizer. SA, compared with JA, had a higher correlation with insect community diversity, dominance index (*D*), and prevalence of Asian corn borers under both normal and reduced fertilizer. It is speculated that intercropping corn increases insect diversity and insect species of natural enemies by decreasing SA concentration and increasing AA content, thus reducing the prevalence of Asian corn borers.

## Discussion

In the present experiment, the investigation of insects on corn plants in the field showed that fertilizer and intercropping types had little effect on the *S*, *H*, *E*, and *D* of insects. Intercropping increases vegetation types and insect habitat diversity. This makes it suitable for more insects to inhabit, making the *S*, *H*, *and E* of insects increase in the field and *D* decrease. The increase may be due to landscape diversity, which attracts more insects, especially the natural enemy insects, and is consistent with Songa et al.'s (2007) hypothesis. In addition, the number of Asian corn borers, a major pest on corn plants, was significantly reduced under intercropping conditions, similar to Ju et al. (2019) and Ouyang et al. (2020). Through the analysis of insect survey data, we found that the number of natural enemy insect species on corn plants increased under intercropping, again supporting Songa’s hypothesis (Songa et al., 2007). Based on data from both years, it was found that the numbers of insect species, Asian corn borers, and natural enemy species (Figure 1, 5, 6) were different between the 2 years and may have been caused by climate differences between years. For example, more or less precipitation will affect the insect community structure in the native area (Menéndez et al., 2007; Warne et al., 2010; Zhu et al., 2014). From the comparison of meteorological data in Jiyang District in Table 1, it can be found that there was a great difference in rainfall between the experimental crop growth periods (from June to October) in 2018 and 2019. The rainfall in 2018 was generally higher than in 2019, which may be the reason why the insect occurrence in 2018 was higher than in 2019. However, there was no significant difference in average temperature between the 2 years. This experiment can verify the view in ecology that intercropping planting increases the diversity of field landscape and attracts more natural enemies, thus effectively reducing the density of insect pests. Intercropping is an effective method of ecological pest control in agricultural production.

Overall, the contents of total nitrogen and total phosphorus in corn leaves of corn–soybean intercropping were significantly higher than those in corn monoculture and corn–millet intercropping. They were also higher than those in corn–peanut intercropping, but the differences were insignificant. Intercropping with legumes can increase the content of N and P in gramineous crops, which is consistent with previous research results (Li et al., 2003; Dahmardeh et al., 2010; Latati et al., 2016). As the row ratio of corn intercropping decreased, the total carbon content in corn leaves decreased and total phosphorus content increased. This indicates that the higher the proportion of nitrogen-fixing crops in the intercropping mode, the higher the content of P. This is consistent with the research results of Dahmardeh et al. (2010). At the same time, Table 4 shows that the main nutrients in corn leaves under both normal fertilizer and reduced fertilizer application in C2S4 were the highest, and previous studies have also confirmed this (Hepperly et al., 2009). Therefore, it is suggested that this model can be promoted in field production to improve crop quality. Other studies have come to similar conclusions (Zhang et al., 2015). Intercropping between gramineae and legumes improves the absorption and transformation of nutrients and increased crop yield (Hinsinger et al., 2011; Latati et al., 2016). In addition, corn intercropping with soybean and peanut increased nitrogen content in corn leaves. Compared with the previously published results (Li et al., 2022), the variation trend of nitrogen content in soil and leaves is consistent under intercropping and fertilizer reduction. Nitrogen is an important element in synthesizing nutrients. The results showed that the change in nitrogen content was consistent with the trend of the three nutrients and consistent with the corresponding corn yield. Therefore, it can be inferred that intercropping and fertilizer reduction affect the uptake of nutrients in corn roots, resulting in yield differences, which is consistent with the results of Li et al. (2022) previously published. Correlation analysis showed that with the increase of nitrogen, the number of Asian corn borers also showed a decreasing trend, which was similar to the results of previous studies (Klostermeyer, 1950). This study supports this conclusion to some extent.

JA and SA have been widely studied in plants and play important roles in plant defense signal transduction. According to the analysis of JA and SA under different treatments and under the same planting pattern, fertilizer reduction increased the content of these two plant hormones. They play an important role in regulating stress resistance, including the insect resistance of corn. However, for JA, we found that the fertilizer level had no significant effect on JA content for corn–soybean and corn–peanut intercropping. This is suspected to be due to the nitrogen fixation capacity of soybeans and peanuts which increased the soil nitrogen fertilizer and significantly reduced the impact of fertilizer reduction.

From the correlation analysis, it can be concluded that *H* and *E* will increase when *S* increases, and of course, NEI will also increase. The increase of *S* promoted stability in insect communities. It simultaneously decreased the Simpson dominance index, *D*, which resulted in a decline in the number of Asian corn borers, a major corn pest. The increase in N and P (Table 6) in leaves is beneficial to the synthesis of leaf nutrients, making more insects benefit and promoting insect communities’ stability. JA and SA, as key hormones in the synthesis of secondary defenses, play a key role in corn’s insect resistance. At the same time, their synthesis is closely related to the nutrient elements. N and P can promote JA synthesis, while C and K are conducive to SA synthesis. There is antagonism between JA and SA. The content of JA and SA is low under normal circumstances, but the increase of N and P is conducive to the synthesis of JA, and the increase of C and K (Table 6) is conducive to the synthesis of SA. *S*, *H*, *E* and NEI were significantly negatively correlated with the prevalence of the main pest, Asian corn borer, under normal or reduced fertilization (Figure 7). In present study, the number of the Asian corn borers decreased when *S*, *H*, *E*, and NEI increased under intercropping conditions. That is, intercropping may reduce the population of Asian corn borers by increasing *S*, *H*, *E*, and NEI.

## Conclusion

**Figure 8:**
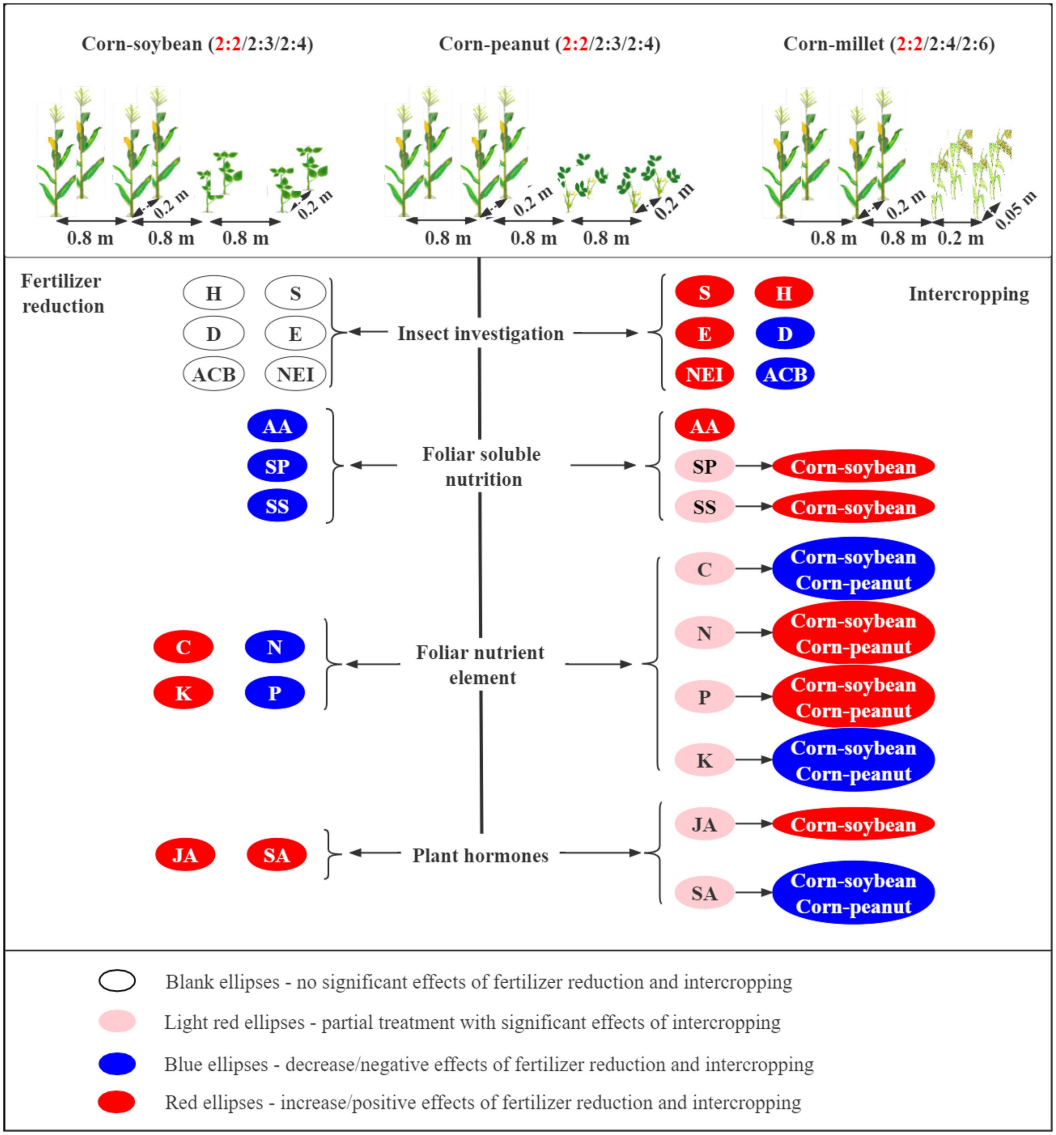
Patterns of intercropping type and fertilizer levels affecting corn nutrients and insect communities (*S*-Species number; *H*-Shannon-Wiener diversity index; *E*-Pielou evenness index; *D*-Simpson dominance index. ACB: number of Asian corn borers; NEI: number of natural enemy insect species; AA: amino acid; SP: soluble protein; SS: soluble sugar; C, N, P and K: total carbon, total nitrogen, total phosphorus and total potassium in corn leaves; JA: jasmonic acid; SA: salicylic acid; →: represents a range from large to small). The effects of fertilizer reduction and intercropping type on corn nutrition and insect communities are shown on the lower left and lower right of the figure, respectively.

This 2-year field experiment was conducted to investigate the effects of corn intercropping types (corn–soybean/corn–peanut/corn–millet) and planting patterns (different row ratios) on corn pests under different fertilizer levels (Figure 8). We found that fertilizer reduction did not significantly influence the insect diversity index and the numbers of the main pest for corn plants. However, compared with corn monoculture, intercropping significantly increased the diversity of species (*S*), Shannon-Wiener diversity index (*H*), and Pielou evenness index (*E*) and significantly reduced the Simpson dominance index (*D*) and the number of Asian corn borers. In addition, the reduction in fertilizer significantly decreased nutrient (amino acid, soluble protein, and soluble sugar) contents in the leaves of corn. Compared with corn monoculture, intercropping significantly increased AA content. corn–soybean intercropping significantly increased the content of SP and SS. In general, the contents of the three nutrients were highest in corn–soybean intercropping at normal fertilizer levels. In addition, reducing fertilizer application was beneficial to total carbon and total potassium accumulation, while corn–soybean and corn–peanut intercropping were beneficial to total nitrogen and total phosphorus accumulation. Overall, reduced fertilizer application was beneficial to the accumulation of total carbon and total potassium in corn. Corn–soybean intercropping under normal fertilizer was beneficial to total nitrogen and total phosphorus accumulation, followed by corn–peanut intercropping. For plant hormones, fertilizer reduction and intercropping increased JA content, among which the JA content was the highest under fertilizer reduction and corn–soybean intercropping. Intercropping was not beneficial to SA synthesis, especially corn–soybean intercropping and corn–peanut intercropping. It was speculated that intercropping corn could increase the species and diversity of natural enemies and reduce the number of Asian corn borers by decreasing SA concentration and increasing AA. The present study shows that corn intercropping could increase insect diversity and reduce the population density of major pests through ecological regulation. In addition, it can increase the utilization and absorption of corn nutrients and indirectly improve the insect resistance of corn plants. The corn–soybean intercropping pattern was the best among the three types of corn intercropping. Finally, the C2S4 pattern was found to be the best.

## Data availability statement

The raw data supporting the conclusions of this article will be made available by the authors, without undue reservation.

## Author contributions

LL, GX, and FC: conceptualization and method. LL, RD, RL, YZ, and JL: data collection. LL: data analysis. LL, GX, and FC: data presentation, writing, reviewing, and editing. All authors contributed to the article and approved the submitted version.

## Funding

This research was funded by the National Key Research and Development Program of China (2021YFD1201604, 2017YFD0200400 and 2021YFF1001204) and the Jiangsu JCIC-MCP Program.

## Conflict of interest

The authors declare that the research was conducted in the absence of any commercial or financial relationships that could be construed as a potential conflict of interest.

## Publisher’s note

All claims expressed in this article are solely those of the authors and do not necessarily represent those of their affiliated organizations, or those of the publisher, the editors and the reviewers. Any product that may be evaluated in this article, or claim that may be made by its manufacturer, is not guaranteed or endorsed by the publisher.
